# Host Immune Response to Bovine Viral Diarrhea Virus (BVDV): Insights and Strategies for Effective Vaccine Design

**DOI:** 10.3390/vaccines13050456

**Published:** 2025-04-25

**Authors:** Asamenew Tesfaye Melkamsew, Tesfaye Sisay Tessema, Jan Paeshuyse

**Affiliations:** 1 Laboratory of Host-Pathogen Interaction in Livestock, Department of Biosystems, Division of Animal and Human Health Engineering, KU Leuven University, 3000 Leuven, Belgium; 2 Unit of Health Biotechnology, Institute of Biotechnology, Addis Ababa University, Addis Ababa P.O. Box 1176, Ethiopia; tesfu74@yahoo.com

**Keywords:** BVDV, BVD, immunity, maternal antibody, vaccines

## Abstract

Bovine viral diarrhea (BVD) is caused by bovine viral diarrhea virus (BVDV), a member of the genus Pestivirus and in the family Flaviviridae. According to some studies, the disease incurs USD 1.5–2.5 billion per year and USD 0.50 to USD 687.80 per cow loss in beef and dairy farms, respectively. Using vaccines is among the strategies to prevent the disease. However, complete protection requires vaccines that target both the humoral and cellular immune responses of the adaptive immune system. A comprehensive literature review was made to provide insights into the interaction of BVDV with host immunity, vaccine applications, and the limitation of the currently available vaccines, as well as explore strategies used to advance the vaccines. BVDV causes immunosuppression by interfering with the innate and adaptive immune systems in a manner that is species and biotype-dependent. Interferon production, apoptosis, neutrophil activity, and antigen-processing and presenting cells are significantly affected during the viral infection. Despite maternal antibodies (MatAbs) being crucial to protect calves from early-age infection, a higher level of MatAbs are counterproductive during the immunization of calves. There are numerous inactivated or modified BVDV vaccines, most of which are made of cytopathic BVDV 1 and 2 and the BVDV 1a subgenotypes. Furthermore, subunit, marker, DNA and mRNA vaccines are made predominantly from E2, E^rns^, and NS_3_ proteins of the virus in combination with modern adjuvants, although the vaccines have not yet been licensed for use and are in the experimental stage. The existing BVDV vaccines target the humoral immune system, which never gives the full picture of protection without the involvement of the cell-mediated immune system. Several limitations were associated with conventional and next-generation vaccines that reduce BVDV vaccine efficiency. In general, providing complete protection against BVDV is very complex, which requires a multi-pronged approach to study factors affecting vaccine efficacy and strategies needed to improve vaccine efficacy and safety.

## 1. Introduction

Bovine viral diarrhea (BVD) globally affects over 80% of the cattle population and negatively affects the economic return from the livestock sector [1,2]. Bovine viral diarrhea virus (BVDV) is a member of the genus Pestivirus in the family Flaviviridae. Three species of the virus are assigned in the genus, including BVDV 1, which has 21 sub-genotypes (1a-1u); BVDV 2, which has four sub-genotypes (2a-2d); and four Hobi-like (BVDV-3) species (3a-3d) [3]. Based on the cytopathic effect on cell lines, the virus is also classified into two biotypes: cytopathic (cp) and non-cytopathic (ncp) BVDV [4]. BVD is characterized by diarrhea, respiratory disease, immunosuppression, abortion, congenital malformations, and giving birth to persistently infected calves (PI) [2].

According to Hilleman [5], viruses employ two different strategies to remain allied with their hosts: (1) a “hit-and-run” strategy characterized by the sequential spread of infection in a series of hosts; (2) a hit–stay or infect and persist strategy that enables the virus to remain in the individual host for an extended period and occasionally or rarely transmit to subsequent hosts. BVD in adult cattle is commonly characterized by transient infection, reflecting the virus’s use of a ‘hit-and-run’ strategy. Conversely, the “hit-stay or infected and persist” strategy is typically observed in calves that are persistently infected with the virus. Hit-and-stay viruses use strategies such as sequestration, the blockade of antigen presentation, cytokine escape, the evasion of natural killer cell activities, escape from apoptosis, and changing the antigenicity as a means to evade the immune system of the host [5].

The innate immune system acts as the first line of defense during viral infections, aiming to prevent viral invasion or replication before the adaptive immune system elicits more specific protection [6]. BVDV impairs both the adaptive and innate immune systems in a manner that is strain and biotype-dependent [7]. It is suggested that BVDV can maintain prolonged infection in immune-privileged sites of the hosts, such as the ovaries, testes, brain, and the circulating white blood cells [8].

The prevention and control of BVD depend on detecting and eliminating PI animals from the herd, surveillance, biosecurity, and effective vaccines [9]. However, there are several limitations in the existing conventional vaccines, such as variation in vaccinal and field strain, limited cross-protection among genotypes and subgenotypes, safety, immunosuppression, reversion to virulence, the induction of neonatal pancytopenia, production cost, repeated injection, and neutralization by MatAbs [10,11,12]. Brackenbury et al. [13] suggested that the host immune response towards pathogens plays a pivotal role in improving vaccine efficacy, including BVDV vaccines.

The existing literature offers limited insight into the correlation between the BVDV vaccination and the host immune response. Moreover, current strategies for advancing BVDV vaccines are inadequately described, and the commercial vaccines discussed lack comprehensive coverage. This literature review presents relevant insights into the host immune response during BVDV infection, strategies aimed at enhancing the efficacy and safety of BVDV vaccines, and an overview of currently available commercial BVDV vaccines. A comprehensive understanding of the virus’s impact on the host immune system helps mitigate the risk of vaccine failure and supports the selection of appropriate BVDV vaccines within vaccination programs.

## 2. Methodology

A comprehensive literature review was conducted following the methods of Williams [14] to provide a detailed overview of BVDV interaction with the host immune system and strategies employed to improve the existing BVDV vaccines. This review is guided by the questions of how BVDV interacts with the host immune system and what vaccine design strategies are available to overcome the limitations of existing conventional and next-generation vaccines. The scope of this review is limited to studies published in English between 2005 and 2024 on topics related to host-immune response against BVDV, BVDV vaccines, and vaccine design strategies. A broad literature search was conducted across multiple electronic databases, including PubMed, Scopus, Google Scholar, ScienceDirect, MEDLINE, and Embase using the search terms “BVD”, “BVDV”, “Bovine viral diarrhea virus”, “Biotypes”, “Bovine”, “Cattle”, “Vaccines”, “Conventional vaccines”, “Cell-mediated immunity”, “Adaptive immunity”, “Humoral immunity”, “T-lymphocytes”, “B-lymphocytes”, “Innate immune cells”, “Innate immune response”, “Maternal Antibody”, “Persistently infected calf”, and “subunit vaccines” combining the Boolean operators “OR” and “AND”. Articles were selected based on their significance to the research question and contribution to understanding the complexity of BVDV interaction with the host immune system and the existing strategies to advance the vaccine. Titles and abstracts were initially selected based on predefined inclusion and exclusion criteria, followed by a thorough review of the full texts to verify the relevance and adequacy of the data for inclusion in the review. Overall, 346 articles were retrieved from the databases, of which 86 were included in this review manuscript. The analysis was conducted using a thematic approach, identifying key topics related to BVDV interaction with the host immune system and the nature of the BVDV vaccines. Reviews, reports, books, seminars, and non-full-text studies and publications in a language other than English were excluded from the review.

## 3. BVDV and the Innate Immune System

### 3.1. Interferon α/β

The induction of alpha/beta interferon (interferon α/β) is the first line of defense against acute viral infection. It has a key role in linking the innate and adaptive immune systems. This multifaceted cytokine has different effects, such as the inhibition of viral replication, the induction of natural killer (NK) cells and macrophages, the increased expression of Major histocompatibility I (MHC I), and the activation of T-cells [15].

A pathogen-infected cell produces interferon type 1, which binds to the receptors of infected and uninfected cells to induce an antiviral effect [16]. Type 1 interferons activate the synthesis of more than 100 cellular proteins. The long-term co-evolution of the host and pathogens allows many viruses to evade the antiviral state of the interferon α/β system [17]. However, some viruses prevent the action of interferon by inhibiting the binding to receptors, hindering the signal transduction pathway, or preventing the antiviral activity of IFN-induced proteins [16].

The single-stranded ribonucleic acid (ssRNA) of both biotypes (ncp and cp) of BVDV blocks the induction of IFN-α/β [18]. Resistance to IFN-α/β depends on the biotypes of the BVDV virus infecting the host.

**Figure 1:**
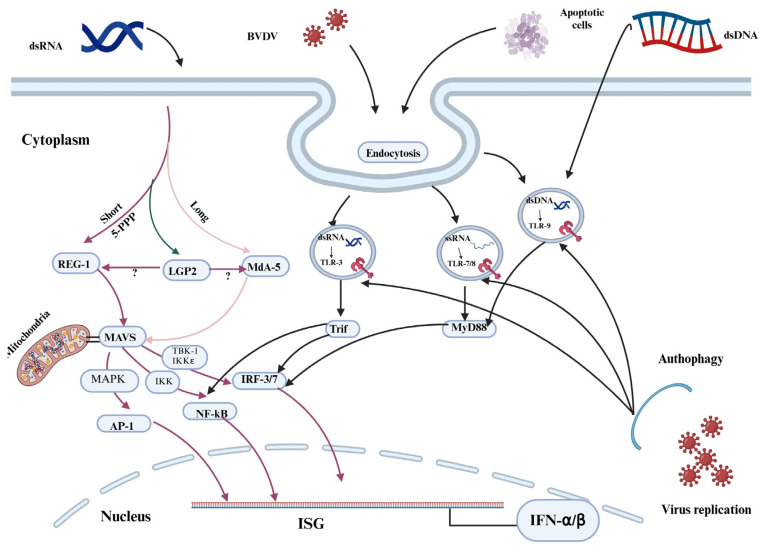
Signaling pathways for Retinoic Acid-Inducible Gene-I-like Receptors (RLRs) and Toll-like receptors (TLRs). The ssRNA of the cpBVDV biotype stimulates endosomal TLR 7/8 to induce interferon-β (IFN-β). As a result, the TRIF temporarily binds with TLR 7/8. After detaching from the receptor, the TRIF forms a speckle-like complex that relocates to interact with downstream signaling molecules such as tumor necrosis factor (TNF) receptor-associated factor 2 (TRAF2), TRAF6, and receptor-interacting protein 1 (RIP-1) [19,20]. Then, the combined effect of TRIF with TRAF3, TRAF family member-associated nuclear transcription factor-κB (NF-κB) activator (TANK)-binding kinase 1 (TBK1), inhibitor of κB (IκB) kinase-related kinase-ε (IKK-ε; also called IKK-i), and NF-κB-activating kinase (NAK)-associated protein 1 (NAP1) can be observed. Finally, the signaling pathways stimulate transcription factors, namely, IFN-regulatory factor 3/7 (IRF3/7), the TRIF-dependent NFκB, and the activator protein 1 (AP-1), thus moderating the production of type I IFNs, proinflammatory cytokines, and chemokines, respectively. AP = Activator Protein; IRF = IFN regulatory factors; IKKε = inhibitor of nuclear factor kappa-B kinase ε; ISG = IFN-stimulated genes; LGP2 = laboratory of genetics and physiology 2; MAVS = mitochondrial antiviral signaling protein; MDA5 = melanoma differentiation-associated protein 5; MAPK = mitogen-activated protein kinase; MyD88 = myeloid differentiation primary response; NF-KB = nuclear factor-kappa B; Tbk = TANK-binding kinase; Trif = TIR domain-containing adapter inducing IFN-β. Created with BioRender.com.

The success of persistent infection or immunotolerance depends on the highly selective “self” and “non-self” model of evasion of the interferon defense system by BVDV during the early stage of fetal development (40–125 days) [7,19]. Daniel et al. [17] suggested that the increased amount of ssRNA produced by cp BVDV has been postulated to stimulate IFN production via the Retinoic Acid-Inducible Gene I (RIG-I) and Toll-like receptor 7/8 (TLR–7/8)-mediated activation of Interferon Regulatory Factor 3 (IRF-3) (Figure 1).

On the contrary, the N-terminal protein fragment of ncpBVDV inhibits the activity of interferon regulatory factor 3 (IRF3) and interferes with the production of IFN-α/β [20,21]. During early infection with ncpBVDV, IRF3 is relocated from the cytoplasm to the nucleus. However, it is blocked from binding to the interferon gene promoter in the nucleus. Subsequently, it blocks interferon synthesis by inhibiting IFN-α/β gene transcription [22]. The N^pro^ results in the polyubiquitination and subsequent destruction of the regulatory factor by cellular multi-catalytic proteasomes in the cytoplasm [22,23]. NCP BVDV can induce complete mitophagy; however, it also inhibits the expression of type I IFNs, inflammatory cytokines, and the apoptotic pathway [24].

The N^pro^ and E^rns^ antagonize type I IFN production in BVDV-infected and non-infected cells, respectively [21,25]. As a consequence, the innate immune system of the host is inhibited [25]. PI calves are established due to the inhibition of type I IFN signaling pathways by the virus [7,25,26,27]. Remarkably, unlike the cp BVDV biotypes, the ncp BVDV biotypes never induce type I interferon in cultured bovine macrophages [28,29].

Cells infected with ncpBVDV biotype become tolerant to potent interferon inducers such as dsRNA, which signals the existence of viral replication in the infected cells [28]. IFN-tau (IFN-τ) upregulates the expression of interferon-stimulating genes (ISGs) in the endometrium of the uterus, which is crucial also for maintaining pregnancy in ruminants. In contrast, the expression of ISGs is downregulated during acute infection with ncp BVDV. Consequently, the virus persists in the host without being effectively cleared by the immune system [16,30]. IFN-τ is known to have antiviral activity similar to the other type I IFNs, and it may well act to prevent the embryo from infection [28].

### 3.2. BVDV Triggers Apoptosis

Apoptosis in infected cells is triggered by the dsRNA of cpBVDV. Grummer et al. [31] reported that cpBVDV disrupts mitochondrial membrane potential (Dcm), promotes cytochrome c translocation to the cytosol, increases caspase-9 activity, and induces overexpression of apoptotic protease-activating factor 1 (APaf-1). Hence, the virus triggers the intrinsic pathway of apoptosis. Levels of endoproteases, including caspase-3 and caspase-9, were significantly higher in cases of mucosal disease (MD). In contrast, in one experimental study, the expression of caspase-8 was reported to be low in both MD cases and control groups [32]. Reflecting that the intrinsic apoptosis pathway is more critical in the pathogenesis of MD than the extrinsic pathway.

### 3.3. BVDV and Innate Immune Cells

The innate immune response is critical for the body’s defense against pathogens, and it is induced when there is an interaction between a pathogen and different receptors, including damage-associated molecular patterns (DAMPs) [33]. The host’s innate immune response primarily produces a non-specific, rapid defense against pathogens through the complement system, natural killer cells, acute-phase protein response, and IFN-α/β. Neutrophils, among the white blood cells, play a key role in the innate immune response [34].

Activated innate immune cells, such as neutrophils, monocytes, macrophages, or endothelial cells, express S100A9, a member of the damage-associated molecular patterns (DAMPs) protein family that initiates its effect through binding to the TLR4/MyD88 pathway [33]. The binding of N^pro^ to S100A9 suppresses the host’s innate immunity response; therefore, it enhances viral replication in the infected cells [33]. The impaired innate immune system due to BVDV infection predisposes animals to concurrent infections with other pathogens [25].

#### 3.3.1. Neutrophils

**Figure 2:**
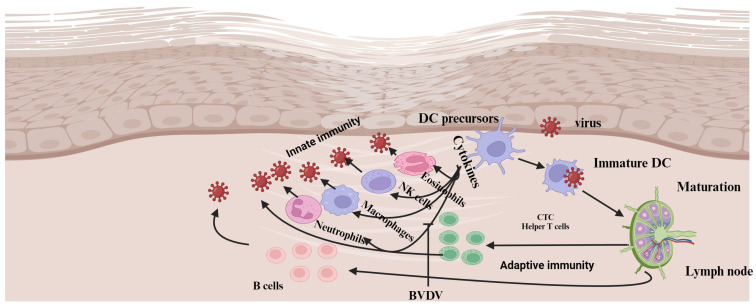
Model for the interaction between the innate and adaptive immune system and BVDV biotypes. Infection of mucosal or epithelial surfaces with cpBVDV activates cytokine expression, including IFNα from different cell types and dendritic cells (DCs). These cytokines induce effector cells of the innate immune response, such as eosinophils, macrophages, and natural killer cells (NK cells). Subsequently, viral replication is blocked due to the combined effect of cytokines and effector cell function. Infection with cpBVDV creates an environment that facilitates the migration of antigen-capturing DCs to the lymph nodes, where they undergo maturation. DCs probably withstand the lytic effect of cpBVDV and migrate to the local lymph node. The DCs with the antigen arrive at the lymph node and present the antigen through MHC I to antigen-specific lymphocytes. Eventually, the activated T lymphocytes (CTLs and helper T cells) migrate to the site of infection to eliminate the virus or virus-infected cells. Mature plasma cells are generated from activated B cells that reach the GC or far in the lymph node and produce virus-neutralizing antibodies. Unlike cpBVDV infection, ncpBVDV infection cannot stimulate an early cytokine response. Consequently, the viral replication is unlimited, and activation of DCs is reduced. DCs, whether they have captured the virus or not, migrate to the local lymph node. Those that encounter the virus within the lymph node produce large quantities of IFNα. The concentration of IFNα in the circulation and tissues increases, ultimately activating the DCs to inhibit viral replication. However, during this time, the ncpBVDV virus has already disseminated throughout the animal body [13]. DCs, dendritic cells; NK cells, natural killer cells; MHC I, major histocompatibility; CTLs, cytotoxic T lymphocytes; GC, germinal center; CD, cluster of differentiation; NET, neutrophil extracellular traps. Created with BioRender.com.

The oxidative burst and neutrophil extracellular activity, as well as the expression of CD18 and L-selectin on neutrophils, are significantly decreased in association with BVDV biotypes [35] (Figure 2). The phagocytic activity of neutrophils is reduced depending on the biotype of the BVDV; for instance, cpBVDV significantly reduces phagocytosis, while ncpBVDV does not [35,36]. Similarly, the expression of CD14 on neutrophils is enhanced by ncpBVDV compared to cpBVDV biotypes. However, the cpBVDV does not have any effect on the chemotactic activity of neutrophils [35].

The activity of neutrophils to migrate towards specific chemical signals and eliminate microorganisms entering the body is determined by the expression of surface markers such as CD14, CD18, and L-selectin [35]. Bovine viral diarrhea virus (BVDV) has been found to compromise neutrophil functions in a biotype-dependent manner [35,36]. The production of nitric oxide and the induction of neutrophil extracellular traps (NETs) are downregulated during infection with cpBVDV biotype [36].

#### 3.3.2. Macrophages

Macrophages play a key role in triggering the innate immune response. Furthermore, they are also crucial in the fight against viral infection. Specifically, macrophages defend indirectly in a biotype-dependent manner during BVDV infection [37]. In the case of persistent infection, macrophages infected with ncpBVDV fail to induce type I IFN. The decreased production of TNF-α by BVDV-infected macrophages derived from bovine bone marrow suggests the suppression of proinflammatory cytokine production [38].

**Figure 3:**
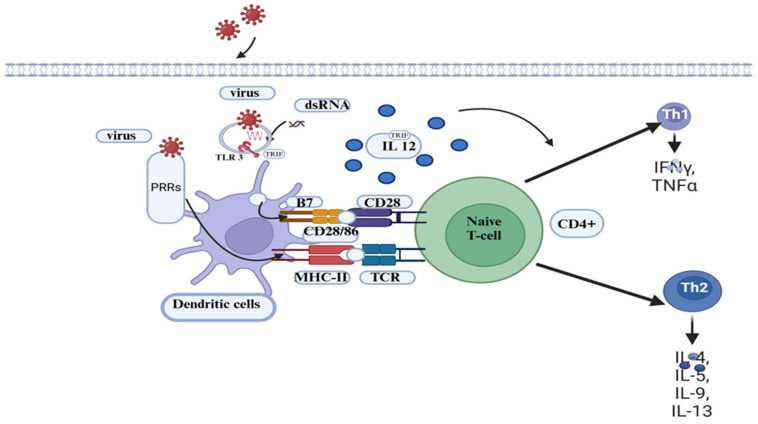
Antigen-presenting cell activation and interactions with T cells. On antigen-presenting cells (e.g., DCs), the viral PAMPs bind to PRRs. Matured and activated DCs migrate with the carried antigen to lymph nodes, where they encounter naïve T lymphocytes and present the processed peptides loaded onto MHC II to CD4+ cells. These CD4+ cells then differentiate into Th1 and Th2 cells in the presence of cytokines and interleukins. Th1 cells activate macrophages to phagocytize and kill the pathogen, and helper B cells produce antibodies against the pathogen. In viral infections such as BVDV, the activation of cytotoxic T cells (CTL) by CD4+ cells plays a crucial role in mounting an effective immune response. DCs with the processed peptides on MHC-I migrate to lymph nodes and bind to naïve CTLs. This activated CTL releases effector molecules such as perforin, granzymes, and granulysin to kill virus-infected cells. PRR, Pathogen Recognition Receptor; PAMP, Pathogen-Associated Molecular Patterns. TLRs, Toll-like receptors; B7/CD80/CD86 represents APCs co-receptor; CD28 denotes T cell co-receptor; and MHC II, Major Histocompatibility II. Created with BioRender.com.

BVDV infection causes immunosuppression and alters the pathogen recognition the receptor (PRR) and Toll-like receptor (TLR) signaling pathway in macrophages (Figure 3). Schaut et al. [39] showed that the expression of pro-inflammatory cells differs between BVDV 2-infected and non-infected macrophages in a manner that depends on both species and biotypes of BVDV. Generally, BVDV-2-infected macrophages are known to impair the production of pro-inflammatory cytokines in response to ligation with TLR, except for TLR7 [40].

Lee et al. [41] suggest that cpBVDV interferes with antigen presentation to immune-competent T cells, while monocyte activation and differentiation are enhanced. As a result, macrophage-mediated uncontrolled inflammation increases viral spread and inhibits the host’s antiviral defense mechanism

#### 3.3.3. Antigen Processing and Presenting Cells (APCs)

The cpBVDV biotype reduces the ability of monocytes to phagocytize antigens and stimulate allogeneic and memory CD4+ T-cell responses [25,41]. Lee et al. [41] indicated that, following BVDV infection, there are proteins involved in the immune responses, such as cell adhesion, apoptosis, antigen uptake, processing, and presentation, and acute-phase response proteins, MHC class I- and II-related proteins, and other molecules involved in the immune function of professional antigen presentation are critically affected by the virus (Figure 3).

The comparison made by Rajput et al. [42] revealed that the expression of surface markers is crucial for immune response activation during infection with pathogens. MHC I, MHC II, and CD86 expression levels in monocyte-derived dendritic cells (Mo-DCs) decreased in ncpBVDV-infected cells while increasing in cells infected with cpBVDV biotypes. Accordingly, BVDV-virus-infected DCs may have a detrimental effect on the host’s immune response [42]. In contrast to this finding, Glew et al. [43] suggested that BVDV does not affect DCs’ ability to present antigens to T cells in vitro; however, it can induce apoptosis in cells infected by the virus. Although BVDV infection does not affect DCs, the virus exploits these cells to spread from the site of infection to other parts of the body. Moreover, infected DCs play a vital role in triggering the immune response towards the virus [44].

## 4. Adaptive Immune System

BVDV causes immunosuppression by disrupting the adaptive immune response, the host’s critical second line of defense against invading pathogens [7]. B- and T-cell immunotolerance has been retained by animals persistently infected with BVDV in a biotype-specific manner [25]. After seven days of infection with cpBVDV, the amount of antibody produced by B cells decreases compared to ncpBVDV infection. The ncpBVDV biotype induces high levels of IgG2 antibody isotype due to the polarization of the immune response towards the Th1 response [7,45]. However, infection with the ncpBVDV impairs the cytotoxic T-lymphocyte response [7]. The adaptive immune system of PI animals remains normal, except for the specific viral strain to which they are immunotolerant. The level of gamma delta T-cells may be related to mucosal disease [7].

According to Peterhans and Schweizer [25], ncpBVDV significantly induces the production of homologous and heterologous neutralizing antibodies compared to cpBVDV infection. However, the production of IgG1 antibody isotype is suppressed significantly during BVDV infection [45]. Depending on viral strains and biotypes, the adaptive immune system is critically affected by the virus [30]. In persistently infected (PI) animals, the strain variation also enables BVDV to evade the adaptive immune system; an immune response targeting one BVDV strain does not prevent the replication of antigenically distinct “non-self” strains, which can infect cells already harboring ncpBVDV [16].

## 5. BVDV and Maternal Antibody (MatAb)

Acquired MatAbs can protect calves from BVDV infection for a limited time, but the high MatAb titers prevent an antibody response following vaccination [46]. The duration of protection provided by MatAbs varies depending on the level of MatAbs and the strain of BVDV infecting the calves. The decrease in the MatAb titer makes calves more prone to viral infection at an early growth stage. Passive antibodies also help to control the spread of viral infection in a group of calves and the accumulation of the virus in the herd by preventing viral shedding from infected or diseased animals [47]. Downey et al. [47] indicated that the MatAb titer level should decline to 3.12 or lower to avoid neutralizing the BVDV vaccine, thereby allowing an effective immune response to be induced in the vaccinated calves. Recent studies showed that breed, parity, and calf gender affect the concentration of immunoglobulin in the colostrum and are also associated with the failure of passive transfer of the antibody [48]. Suggesting that those factors should be taken into consideration during the vaccination program.

## 6. BVDV Vaccines and Application

### 6.1. Conventional Vaccines

Complete protection against BVDV infection depends on the vaccine’s ability to elicit both the humoral and cellular immune responses in the host. Vaccinating dairy and beef animals is required to prevent clinical disease and viral dissemination in the herd [49]. Although BVDV vaccines are effective in reducing disease incidence and viral transmission, several challenges hinder vaccine efficiency. These include genotypes and sub-genotypes variation, the existence of persistently infected cattle, viral tropism for immune cells, limited cross-protection among strains, and concern regarding vaccine safety and efficacy [9,50,51]. A variety of BVDV vaccines are available globally for cattle, most of which are multivalent formulations that combine BVDV with other viral antigens, multiple viral components, and bacterial vaccines, bacterins, or toxoids (Table 1). The BVDV vaccines can be classified as modified live vaccines (MLVs), inactivated, or chemically altered live vaccines.

Under experimental conditions, the efficacy of licensed BVDV vaccines in protecting the dam and fetus ranges from 91% to 100% [51,52]. The effectiveness of the vaccine is critically determined by the duration of exposure to PI animals under field conditions [53]. Grooms et al. [54] revealed that animals immunized with two doses of inactivated vaccine had a 73% protection level for the fetus following 98 days of exposure to wild BVDV viral strains. Rodning et al. [55] measured the efficacy of three BVDV vaccines and found that the vaccines can protect the fetus, even with prolonged exposure to the virus.

Modified live vaccines (MLVs) provide higher protection and a longer-lasting immune response compared to killed vaccines [56,57]. In contrast, inactivated or killed vaccines have low efficiency in protecting the fetus and require higher production costs and higher doses of the antigen during vaccination [58]. Due to safety concerns, farmers prefer killed to modified live vaccines during the vaccination program, especially during the breeding period [59]. Although MLVs are neutralized by a higher level of MatAbs, such vaccines offer better protection than killed vaccines against BVDV infection [11]. Certain BVDV MLV vaccines, such as Bovela^®^ live double-deleted vaccine, can be used during high levels of MatAb titer in calves [60,61].

An effective vaccine candidate should provide cross-protection against the common circulating subgenotypes of BVDV-1 and 2 [62]. Most inactivated vaccines induce antibody production and activate MHC II-restricted helper and/or cytotoxic T lymphocytes (CTL). Nevertheless, they lack the activation of CD8+ MHC I-restricted CTL, which plays a key role during viral infection [58]. The strategy to vaccinate against BVDV is to prevent the vertical transmission of the virus from the dam to the fetus during an early stage of pregnancy. This is to avoid the production of PI calves on the farm [50].

Conventional BVDV vaccines (inactivated and modified live vaccines) contain a combination of BVDV 1 and 2 species (Table 1). Platt et al. [60] revealed that a pentavalent MLV containing genotypes BVDV 1 and 2 induces T-cell responses (CD4+ and CD8+) in calves aged 1–8 weeks in the presence of maternal antibodies. On the other hand, inactivated BVD vaccines elicit a robust humoral response and activate subsets of cell-mediated immunity, including the T helper 1 response, gdTCR+ T cell response, and CD8+ γδTCR+ T cell response. However, this type of vaccine does not induce a detectable CD8+ γδTCR− (cytotoxic T cell) level during an immune response [61].

The antigenicity of the BVDV vaccine is typically assessed using the viral neutralization technique (VNT), which only reflects the humoral component of the immune system. Assessing cell-mediated immunity (CMI) alongside humoral immunity provides a comprehensive understanding of the host immune response to BVDV infection. The vaccine type used at first immunization (live versus killed BVDV vaccines) determines the status of the host immune response. Vaccinating dairy animals initially with live BVDV vaccine, followed by killed vaccine, enhances host CMI and humoral responses [10]. Sozzi et al. [63] reported no strong antibody-mediated cross-immunity against all studied BVDV strains. Moreover, the BVDV vaccine inactivated with hydrogen peroxide demonstrated greater safety and immunogenicity compared to the formaldehyde-inactivated vaccines, as observed in experimental mice [64]. The level of antibody titer after immunization against BVDV is influenced by continued vaccination, viral strains involved, and the age at vaccination [59].

### 6.2. Next-Generation Vaccines

Currently, advanced technologies are employed to produce BVDV subunit, marker vaccines, DNA, and mRNA vaccines using viral structural and non-structural proteins. E2, E^rn^s, and NS_3_ are the immunodominant viral protein structures commonly used to develop BVDV vaccines. The E_2_ protein, a key inducer of neutralizing antibody production, is frequently utilized in the development of subunit, DNA, and mRNA vaccines [65,66]. Conversely, the non-neutralizing inducer E^rns^ and NS_3_ are used to develop marker vaccines (Table 2).

Nelson et al. [72] developed plant-based recombinant tE_2_ vaccines that induce the immune response in guinea pigs against BVDV. Gómez-Romero et al. [65] induced a strong immune response against BVDV using a combination of rE_2_ glycoprotein. E2-based subunit vaccines provide significant protection against BVDV 2; therefore, this structural protein is a better candidate for developing engineered vaccines [73]. Pecora et al. [74] developed a truncated E2 (tE2) vaccine for BVDV 1b and 2a by binding the tE2 version to a single-chain fragment variable (scFV) called APCH to produce APCH-tE2-1a-1b-2a vaccines. The vaccine is safe and induces a rapid and sustained neutralizing antibody response compared to a conventional vaccine in cattle [75]. APCH-tE2, developed using alfalfa transgenic plants, provides complete protection against BVDV [76]. The efficacy of E2 subunit vaccines to protect against BVDV can be enhanced by using adjuvants in combination with the vaccine [76,77].

Furthermore, the use of the Lactobacillus-expressed E_2_ protein, in combination with pPG-E2_ctxB/Lc W56 conjugated to cholera toxin B subunit (ctxB) as an adjuvant, has been employed to enhance the immunogenicity of the vaccines. This vaccine was immunogenic and induced effective mucosal, humoral, and cellular immune responses in experimental mice [68]. Sangewar et al. [11] developed a vaccine called Expi293TM, expressing antigens (293F-E2^123^, 293F-NS2-3^1^, and 293F-NS2-3^2^) that elicited strong and cross-reactive IFN-γ responses and neutralizing antibodies specific to BVDV. A recombinant vaccine using five E2 proteins from BVDV1 subgenotypes was also found to be efficient in inducing the production of neutralizing antibodies against the virus [78].

A mutated BoHV1 vaccine vector expressing chimeric E_2_ and flag-tagged E^rns^, fused with the granulocyte monocyte colony-stimulating factor (GM-CSF), has triggered the production of cross-reactive neutralizing antibodies and a cellular immune response [71]. The Pestivirus E^rns^ gene originated from the pronghorn antelope used to develop a chimeric NADL/P-E^rns^ marker vaccine and used during the BVDV control and eradication program [79]. The fusion vaccine made of E2s and the complement C3d (E2s-C3d) provided sufficient protection against BVDV [80]. Subunit vaccines E2Fc and E2Ft, developed by Yanqing et al. [81], were shown to activate the mucosal immune response, leading to higher IgA titers effective against BVDV infection.

Koethe et al. [69] developed BVDV-1b_synCP7_∆Npro_E^rns^ and Bungo BVDV-1b_synCP7_∆Npro_E^rns^ Bungo_E1E2BVDV-2 CS synthetic chimera vaccines, which are safe for use in cattle and provide effective protection against BVDV. These vaccines also serve as marker vaccines to differentiate between vaccinated and infected animals. An adenoviral vector-based vaccine developed using three novel mosaic polypeptide chimeras, such as NproE2^123^, NS23^1^, and NS23^2^, has also induced higher IFN-γ spot-forming cells, T-cell proliferation, and higher specific antibodies against the BVDV-1 strain [12]. Moreover, the potency of the DNA vaccine for BVDV (NTC E2t(co) and NTC NS3(co)) in cattle has also been augmented by the co-expression of the retinoic-acid-inducible gene I (RIG-I) agonist with the viral antigen [70]. Furthermore, recent mRNA-based BVDV vaccines have demonstrated good safety and immunogenicity during pre-clinical trials in mice [82].

Despite the availability of advanced vaccines against BVDV, several limitations persist, including inadequate activation of humoral and cellular immune responses and adjuvant-related toxicity, which hinders their development and widespread use [81,83]. Although the safety of using DNA and subunit vaccines in animals is questionable, it is still an opportunity to introduce next-generation vaccines to the vaccine market [80]. The use of modern adjuvants such as silica vesicles and hollow-type mesoporous silica nanoparticles (HMSA), as nanocarriers to load E_2_ protein, enhances the induction of cellular and humoral immunity response in the host [81].

## 7. Conclusions

Bovine viral diarrhea negatively affects the financial return from cattle production by increasing morbidity and mortality in dairy and beef farms. The prevention and control of the disease depend on a combination of biosecurity, vaccines, and the detection and elimination of persistently infected calves from the herd. The virus causes immunosuppression by blocking the innate and adaptive immune responses in a strain and biotype-dependent manner. Numerous commercial vaccines are available, including monovalent or polyvalent, modified, or killed vaccines. The main drawbacks of these vaccines are the lack of or minimal cross-protection, high production costs (for killed and subunit vaccines), safety concerns (for MLV vaccines), and a weak activation of both humoral and cellular immune responses. To overcome these limitations, the advancement of vaccines and immunomodulators is mandatory. Most next-generation vaccines are still in experimental trials and are not available in the commercial market. In general, the prevention and control of BVDV are highly complex and necessitate a multi-pronged approach to fully understand factors influencing vaccine efficacy, such as age at vaccination, pregnancy status, vaccine type, and the circulating genotypes and subgenotypes of the virus. We expect this review contribute meaningfully to the ongoing discourse on this important subject.

## Figures and Tables

**Table 1:** Various types of BVD-licensed commercial vaccines are available for use in cattle.

Vaccines Name	Types	Dose (mL)	RA	Booster	Used During Pregnancy	Genotypes	Strains	Subtypes	Biotypes	Manufacturers	Website (Assessed on 10 February 2025)
Bovi-Shield GOLD^®^ IBR-BVD	MLV	2 mL	SC/IM	1 year	No	BVDV1 and 2	NADL	1a	ncp	Zoetis, NJ, USA	https://www.zoetisus.com/products/cattle/bovi-shield-gold-ibr-bvd
Bovela	MLV	2 mL	IM	1 year	Yes	BVDV1 and 2	KE-9 and NY_93	1a/2a	ncp	Boehringer	https://www.boehringer-ingelheim.com/animal-health/products/bovela
Bovidec	IV	4 mL	SC	1 year	No	BVDV 1	KY1203	NA	ncp	Zoetis, NJ, USA	https://vmd.defra.gov.uk/ProductInformationDatabase/files/QRD_Documents/QRD-Auth_1189212.PDF
Bovilis^®^ BVD	IV	2 ml	IM	1 year	Yes	BVDV1	C-86	1a	cp	Zoetis, NJ, USA	https://www.bovilis.com/bovilis-bvd/
ONE SHOT^®^ BVD	IV	2 mL	SC	1 year	No	BVDV1 and 2	NA	NA	NA	Zoetis, NJ, USA	https://www.zoetisus.com/products/cattle/one-shot-bvd
PESTIGARD^®^	IV	2 mL	SC		Yes	BVDV	NA	NA	NA	Zoetis, NJ, USA	https://www.zoetis.com.au/all-products/portal-site/beef-dairy-sheep/beef-pestigard.aspx
BOVI-SHIELD GOLD FP 5	IV	2 mL	IM	1 year	No	BVDV1 and 2	NADAL/53637	1a/2a	cp	Zoetis, NJ, USA	https://www.zoetisus.com/products/cattle/bovi-shield-gold-fp-5-l5
Cattle Master GOLD FP 5	IV	2 mL	SC	1 year	Yes	BVDV1 and 2	5960/53637	1a/2a	cp	Zoetis, NJ, USA	https://www.zoetisus.com/products/cattle/cattlemaster-gold-fp-5
CATTLEMASTER 4	IV	2 mL	IM	1 year	Yes	BVDV1	5960/6309	1a/1	ncp/cp	Zoetis, NJ, USA	https://www.zoetis.co.za/products/beef-and-feedlot/cattlemaster-4.aspx
Elite^®^ 4-HS	IV	5 mL	IM	1 year	Yes	BVDV1	Singer	1a	cp	Boehringer, Ingelheim, Germany	https://store.animalhealthusa.com/elite4hs.html
Master Guard 10 HB	MLV/IV	3 mL	IM/SC	1 year	Yes	BVD1 and 2	C24V	1a	cp	Elanco, Indiana, USA	https://farmanimal.elanco.com/us/brand/master-guard
Triangle 5	IV	2 mL	IM/SC	1 year	Yes	BVDV1 and 2	Singer/5912	1a/2a	cp/cp	Boehringer, Ingelheim, Germany	https://bi-animalhealth.com/cattle/products/triangle
Vira Shield 6 + VL5 HB	IV	5 mL	SC	1 year	Yes	BVDV1 and 2	K12/GL 760/TN131	1a/1a	cp/ncp/cp	Novarties	https://farmanimal.elanco.com/us/dairy/products/vira-shield
Express FP 5	MLV	2 mL		1 year	Yes	BVDV 1 and 2	Singer	1a/2c	cp/ncp/cp	Boehringer, Ingelheim, Germany	https://www.drugs.com/vet/express-fp-5.html
Pyramid 4 cattle vaccine	MLV	2 mL	SC/IM	1 year	No	BVDV1 and 2	Singer	1a	cp	Boehringer, Ingelheim, Germany	https://www.valleyvet.com/ct_detail.html?pgguid=30e076d9-7b6a-11d5-a192-00b0d0204ae5
Titanium 5	MLV	2 mL	SC	1 year	Yes	BVDV1 and 2	C24V/296	1a/2c	cp	Elanco, Indiana, USA	https://farmanimal.elanco.com/us/beef/products/titanium
TRIANGLE^®^ 4 + PH-K	IV	5 mL	SC	1 year	Not Tested	BVD1	Singer	1a	cp	Boehringer, Ingelheim, Germany	https://www.drugs.com/vet/triangle-4-ph-k.html

N.B. RA = routes of administration; cp = cytopathic; IM = intra muscular; IV = inactivated vaccine; MLV = modified live vaccine; ncp = non-cytopathic; NA = not available; SC = sub-cutaneous.

**Table 2:** BVDV next-generation vaccines with modern adjuvants.

Antigen	Adjuvants	Vaccine Types	References
tE2	Montanide ISA 70 SEPPIC^®^ and (Al(OH)3Hydrogel^®^)	Subunit	[67]
rE_2_	ISA 61 VG	Recombinant	[65]
E2	Cholera toxin B subunit (ctxB)	Recombinant	[68]
E2,NS_2-3_	MONTANIDETM ISA 201 VG adjuvant	Subunit	[11]
Erns-E2	MF59 and CPG-ODN	Subunit	[67]
E^rns^	NA	Marker vaccine	[69]
E2 (E2t) and NS3	NA	DNA	[70]
E2 and flag-tagged Erns	NA		[71]
Npro,E2 and NS2-3	BenchMark-Vaxliant	Mosaic vaccines	[11]

N.B. tE2 = truncated Envelope protein 2; NA = not applicable; ISA = incomplete septic adjuvant; CPG-ODN = CpG oligodeoxynucleotides.
